# Association Between the Attention Network Test, Neuropsychological Measures, and Disability in Post-Acute Traumatic Brain Injury

**DOI:** 10.1089/neur.2022.0068

**Published:** 2023-05-15

**Authors:** Abhishek Jaywant, Emily Blunt, Keith Jamison, Nayoung Kim, Arindam RoyChoudhury, Nicholas D. Schiff, Amy Kuceyeski, Kristen Dams-O'Connor, Sudhin Shah

**Affiliations:** ^1^ Department of Psychiatry, Weill Cornell Medicine, New York, New York, USA.; ^2^ Department of Rehabilitation Medicine, Weill Cornell Medicine, New York, New York, USA.; ^3^ NewYork-Presbyterian Hospital, New York, New York, USA.; ^4^ Brain Injury Research Center, Icahn School of Medicine at Mount Sinai, New York, New York, USA.; ^5^ Department of Rehabilitation and Human Performance, Icahn School of Medicine at Mount Sinai, New York, New York, USA.; ^6^ Department of Neurology, Icahn School of Medicine at Mount Sinai, New York, New York, USA.; ^7^ Department of Radiology, Weill Cornell Medicine, New York, New York, USA.; ^8^ Brain and Mind Research Institute, Weill Cornell Medicine, New York, New York, USA.; ^9^ Department of Population Health Sciences, Weill Cornell Medicine, New York, New York, USA.; ^10^ Department of Neurology, Weill Cornell Medicine, New York, New York, USA.

**Keywords:** attention, brain injury, cognition, executive function, neuropsychology

## Abstract

Cognitive impairment after traumatic brain injury (TBI) is persistent and disabling. Assessing cognitive function in a reliable and valid manner, using measures that are sensitive to the integrity of underlying neural substrates, is crucial in clinical research. The Attention Network Test (ANT) is one such assessment measure that has demonstrated associations with neural regions involved in attention; however, clinical utility of the ANT is limited because its relationship with neuropsychological measures of cognitive function (i.e., its construct validity) has not yet been established in TBI. We evaluated the association between the ANT and 1) a neuropsychological battery assessing executive function and memory and 2) global function assessed by the Glasgow Outcome Scale-Extended (GOSE). Forty-eight adults with complicated mild-severe TBI were evaluated ∼5 months post-injury. Using principal component analysis and multi-variate linear regression adjusted for age, gender, education, and cause of injury, we found that ANT reaction time and executive network scores predicted a principal component assessing processing speed and executive function. Conversely, the ANT did not predict a principal component assessing memory. The ANT was weakly associated with the GOSE. Among persons with TBI during the post-acute phase of recovery, the ANT has good construct validity as evidenced by its associations with neuropsychological measures of processing speed and executive function, but not memory. Given that ANT networks are known to relate to specific neuroanatomical regions, the ANT may be a useful outcome measure for evaluating novel therapeutics targeting attention and executive functions after TBI.

## Introduction

Cognitive dysfunction is a common and disabling sequela after traumatic brain injury (TBI).^1,2^ Among cognitive domains, impairments are frequently observed in attention, processing speed, executive functions, and memory.^3–6^ There are currently no disease-modifying therapeutic interventions. Assessing cognitive function in a reliable and valid manner, using tools that are sensitive to underlying brain network changes, is critical to evaluating the efficacy of interventions.

The Attention Network Test (ANT) is a behavioral measure that is potentially well suited to assess attentional impairment after TBI. The ANT was developed based on Posner's seminal model of attention^7^ to assess three relatively distinct networks of attention^8–10^: 1) the alerting network, which allows the person to maintain a state of alertness in preparation to respond to a stimulus, and is assessed in the ANT by change in reaction time (RT) when cued to stimulus versus not cued; (2) the orienting network, which enables the person to attend to the spatial location of an upcoming stimulus, and is assessed in the ANT based on change in RT when cued to location; and (3) the executive network, which allows the person to resolve conflict and inhibit distraction, and is assessed in the ANT by change in RT to trials with low and high distractors using the flanker paradigm.^11^

Converging evidence from structural and functional neuroimaging paradigms suggest that the three attention networks are dissociable. Anatomical imaging has shown cortical thickness in parietal regions to be associated with alerting attention and cortical thickness in the anterior cingulate and lateral pre-frontal cortex to be associated with executive attention.^12^ Structural connectivity assessed using diffusion magnetic resonance imaging and graph measures of local efficiency correlate differentially with executive attention (lateral pre-frontal cortex), alerting attention (thalamus, inferior parietal), and orienting attention (paracentral lobule, occipital region).^13^ Studies using functional magnetic resonance imaging are generally consistent, showing that alerting attention is associated with activation in frontoparietal and thalamic regions; orienting attention with activation in the superior parietal lobe, frontal eye fields, and midbrain; and executive attention with activation in the anterior cingulate cortex and lateral pre-frontal cortex.^14–16^ Graph measures of global and local functional connectivity also differ by attention network.^17^ Interestingly, there is electrophysiological and behavioral evidence that the executive network may be particularly distinct from the alerting and orienting networks.^18–20^

That the ANT shows strong associations with underlying neural substrates, and that it can be administered concurrently with neuroimaging paradigms, affords it an advantage in the armamentarium of cognitive tests available to use in clinical research in TBI. However, to be suitable for clinical research studies and potentially as an end-point in clinical trials, the ANT should ideally demonstrate sound psychometric properties. Investigations into the psychometric properties of the ANT have provided initial support for its reliability. This includes evidence for split-half reliability as well as test-retest reliability over intervals of 1 week and 6 months.^21,22^ In multiple sclerosis, the ANT demonstrates good reliability when administered monthly over 6 months.^23^

Construct validity, an important psychometric property of an assessment measure, has been demonstrated in healthy young and old adults through associations between the ANT and neuropsychological measures.^22,24^ Though the ANT has been used in TBI,^19,25,26^ to our knowledge, the relationship between the ANT and neuropsychological measures in TBI (i.e., its construct validity) has not yet been evaluated; that is, there is a gap in our understanding of whether and how disruption of attentional networks assayed by the ANT is, in fact, related to clinical neuropsychological deficits observed in persons with TBI. Because the construct validity of measures such as the ANT is specific to disease populations, it is important to test whether and how the ANT relates to neuropsychological measures in TBI, which would provide support for the ANT's utility in TBI clinical research.

The primary goal of the present study was to evaluate the relationship between the ANT and neuropsychological measures of cognitive function in persons with TBI with heterogenous pathology across a range of clinical severity. The neuropsychological measures that we included in the study were assessments of processing speed, executive function, and memory. To provide evidence of construct validity, we expected that the ANT would have stronger associations with neuropsychological measures of processing speed and executive functions and relatively weak associations with measures of memory. Among specific networks, we hypothesized that the ANT executive network would show the strongest divergence (i.e., correlation with processing speed/executive function measures and lack of correlation with memory measures) and that this pattern would be less evident in the ANT alerting and orienting networks.

A secondary goal of this study was to determine the association between attention networks assessed by the ANT and global function using the Glasgow Outcome Scale-Extended (GOSE). We conducted this secondary analysis to explore the potential association between the ANT and “real-world” function, to better understand the ecological validity of the ANT. Past research has shown attentional performance on the ANT to correlate with everyday cognitive failures and with driving in healthy adults,^27,28^ but how the ANT relates to function in TBI remains unknown. We hypothesized that worse performance on the ANT, particularly the ANT executive network, would be associated with worse function as assessed by the GOSE.

## Methods

### Participants

We recruited 48 community-dwelling adults with TBI who met the following criteria: ≥18 years of age; English speaking; capable of providing informed consent independently or through a proxy/legally authorized representative; not currently taking any psychoactive medication; no history of schizophrenia or substance- or alcohol-use disorder; no history of epilepsy, stroke, dementia, or other neurological illness by self-report; and not pregnant. Participants in the TBI group were required to have sustained a complicated mild (Glasgow Coma Scale [GCS] score of 13–15 with evidence of intracranial lesion as verified on acute neuroimaging) or moderate-severe TBI (GCS score ≤12) within the last 6 months. All participants had previously been treated at large, urban medical centers in the New York City metropolitan area. All study activities took place at Weill Cornell Medicine and were approved by the Weill Cornell Medicine Institutional Review Board. Informed consent was obtained in writing from all participants before study activities.

### Assessments

#### The Attention Network Test

**FIG. 1.**
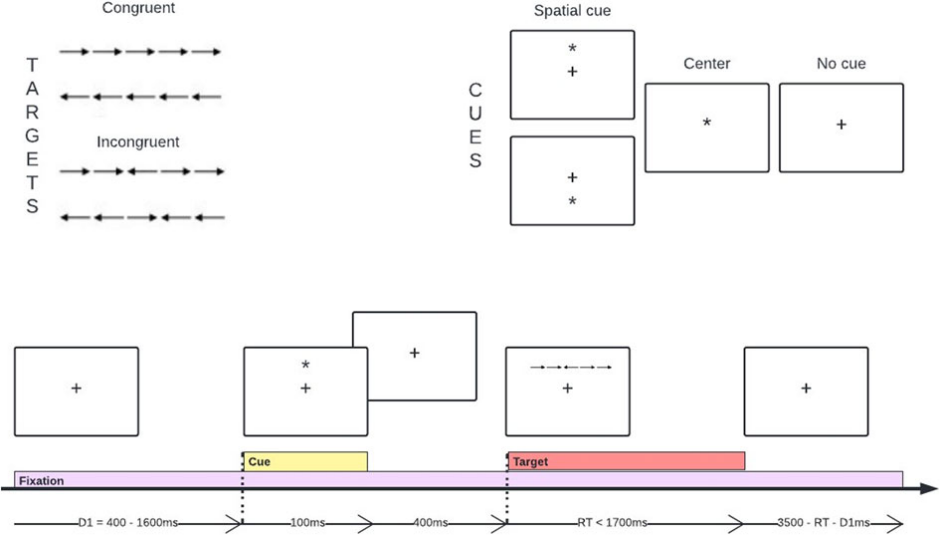
Trial design of the Attention Network Test (ANT). A fixational cross located at the center of the screen is present throughout the test. A cue may appear 500 ms before the target—either above, below, or on top of the fixation point—and last for 100 ms. The target, that is, the center arrow, may be within a congruent or incongruent line of arrows. The target remains on the screen until the subject responds.

During the ANT,^8^ participants view brief presentations of a set of five arrows (Fig. 1). The task is to indicate the direction of the target (center) arrow by pushing the left or right button on a mouse. There are two target conditions: 1) congruent, all five arrows point in the same direction, and 2) incongruent, center arrow is in the opposite direction to flanker arrows. Participants are asked to fixate on a cross on the center of the screen before each trial begins. Three cue conditions precede the target—no cue, center cue, and spatial cue (96 trials each). Center cues alerted participants to the timing of the upcoming target, whereas spatial cues alerted participants to the location (above or below fixation) of the upcoming target. There was a training block of 24 trials with feedback, followed by three blocks of 96 trials each with no feedback. Participants performed the task in a quiet, private room and took brief breaks between the blocks if needed.

We calculated network measures, for correct trials only, by subtracting median RTs between contrasting trials, as follows: 1) alerting = RT_no cue_ −RT_central cue_; 2) orienting = RT_center cue_ −RT_spatial cue_; and 3) executive = RT_incongruent flanker_ −RT_congruent flanker_. Whereas larger alerting and orienting scores indicate faster cue-related performance, larger executive scores indicate worse performance (longer time to resolve conflict). We additionally calculated the mean RT across the 288 trials.

#### Neuropsychological assessment

The neuropsychological assessment battery encompassed measures of attention, processing speed, executive function, and memory and included: the Wechsler Adult Intelligence Scale–Fourth Edition (WAIS-IV) Digit Span (total correct), Letter-Number Sequencing (total correct), Coding (total correct), and Symbol Search subtests (accuracy); the Trail Making Test-A (TMT-A) and -B (TMT-B; time to completion); the Stroop Color Word Test (number correct on word reading, color naming, and color-word interference); and the California Verbal Learning Test–Second Edition (CVLT-II; number of words recalled on Trials 1–5, short delay free recall, and long delay free recall). We chose these measures because they allowed us to evaluate the specificity of the association between ANT network scores and the cognitive functions most commonly affected in TBI,^3–6^ as well as test the hypothesis that the ANT would be more strongly associated with measures of attention, processing speed, and executive function compared to measures of memory.

#### Glasgow Outcome Scale-Extended

The GOSE is a structured interview designed to assess global function and recovery after a TBI. It is administered as a standardized interview with guided questions designed to assess each domain of functioning (consciousness, function in home, function outside the home, work/study, social and leisure activities, family and friendships, and symptoms) while allowing the examiner to apply his or her clinical judgment. Based on the standardized interview, each participant is assigned a value on an 8-point scale, where higher scores reflect better global function.

### Statistical analysis

Statistical analyses were conducted using SPSS (SPSS, Inc., Chicago, IL) and Jamovi. Demographic and clinical characteristics, including impairment on neuropsychological measures, were evaluated descriptively. We calculated norm-referenced *z*-scores for each participant's performance on each neuropsychological measure using published demographically corrected normative data. For descriptive analyses of the ANT, we analogously calculated norm-referenced *z*-scores for each participant's performance using an unpublished, laboratory-based data set of 66 healthy adults ANT scores (mean age = 43.9 years, standard deviation [SD] = 15.8, range = 22–86). With this data set, we created a regression model with ANT network scores as the dependent variable and mean RT from age as a predictor variable (i.e., ANT predicted score = y + B * age). We calculated *z*-scores for subjects with TBI by subtracting their predicted ANT score from the age-predicted score (using the equation estimated by our regression) and dividing by the SD of the healthy adult database. Signs were flipped for ANT executive and mean RT so that negative values always denote worse performance.

To test our hypotheses, we first conducted a principal components analysis (PCA) on the neuropsychological measures (raw scores) to identify the core constructs assessed in our neuropsychological battery, and mitigate the risk of false-positive results arising from multiple comparisons from analyzing each neuropsychological measure separately. We used the Kaiser-Meyer-Olkin (KMO) test and Bartlett's test of sphericity to determine whether the obtained results met assumptions of PCA. We examined the scree plot and extracted components with eigenvalues >1. We used the varimax rotation to obtain the rotated component matrix. Individual scores for each component for each subject were generated in SPSS using the regression method of computing factor scores.

Separate ordinary least squares linear regression models were used to test the association between ANT variables (alerting network score, orienting network score, executive network score, and mean response time) and PCA component 1 (processing speed and executive function) and PCA component 2 (working and episodic memory). Age, education, cause of injury, and sex were included as covariates. All models were evaluated to ensure that assumptions of linear regression were met, including lack of multi-collinearity, normal distribution of residuals, acceptable number of outliers, and homoscedasticity. A similar model was computed with GOSE as the outcome using ordinal logistic regression.

## Results

### Demographics and clinical characteristics

Of the 48 recruited participants, 1 was excluded from data analysis because they were not able to complete the full neuropsychological battery, 1 because of stimulant use which is an exclusion criterion but was not reported at the time of initial enrollment, and 1 because they performed at chance on the ANT, possibly suggesting poor effort. This resulted in a final analyzed sample of 45. Table 1 provides demographic and clinical characteristics of the final sample used in subsequent analyses. In brief, subjects ranged in age from 19 to 85 years, had an average of 14.8 years of education, and ranged in injury severity from complicated mild to severe.

**Table 1.** Sample Demographics and Clinical Characteristics of TBI Sample (*N* = 45)

Demographics/characteristics	Mean (SD) or* N *(%)
Age (years)	47.5 (16.4)
Education (years)	14.8 (3.3)
Sex Male Female	32 (71%) 13 (29%)
Race/ethnicity White Black Asian/Pacific Islander Hispanic origin Other Declined to report	21 (47%) 8 (18%) 5 (11%) 4 (9%) 4 (9%) 3 (6%)
Initial GCS (median; range)	14 (3–15)
Injury severity Complicated mild (GCS 13–15) Moderate (GCS 9–12) Severe (GCS 3–8)	28 (67%) 5 (12%) 9 (21%)
Cause of injury Vehicular accident Fall Assault Other (e.g., gunshot wound)	17 (39%) 15 (34%) 9 (21%) 3 (7%)
Time post-injury (months)	5.0 (1.3)
GOSE 1: Death 2: Vegetative state 3: Lower severe disability 4: Upper severe disability 5: Lower moderate disability 6: Upper moderate disability 7: Lower good recovery 8: Upper good recovery	0 (0%) 0 (0%) 5 (11%) 7 (15%) 7 (15%) 6 (13%) 11 (24%) 10 (22%)

TBI, traumatic brain injury; GCS, Glasgow Coma Scale; GOSE, Glasgow Outcome Scale-Extended; SD, standard deviation.

### Performance on the Attention Network Test and the neuropsychological battery

**FIG. 2.**
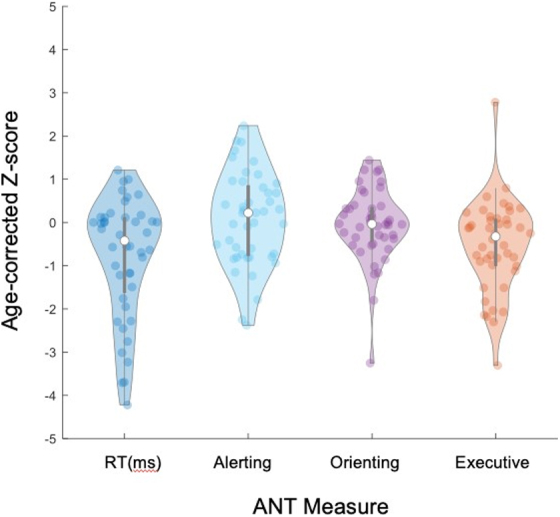
Violin plot demonstrating the distribution of age-corrected z-scores on the Attention Network Test (ANT). Lower (and more negative) z-scores reflect worse performance relative to age expectation.

Participants showed a range of performance on the ANT measures (Fig. 2), with the greatest proportion of scores below age expectations (negative *z*-scores) for mean RT. On neuropsychological measures, we followed consensus guidelines^29^ and defined an average or above score (collapsed into one group for simplicity) as a norm-referenced *z*-score of >−0.67 (≥25th percentile), a low average score as a norm-referenced *z*-score ranging from −0.67 to −1.33 (9–24th percentile), a below-average/borderline score as a norm-referenced *z*-score ranging from −1.22 to −2.25 (2–8th percentile), and an exceptionally low score as a norm-referenced *z*-score <−2.25 (<1st percentile).

**FIG. 3.**
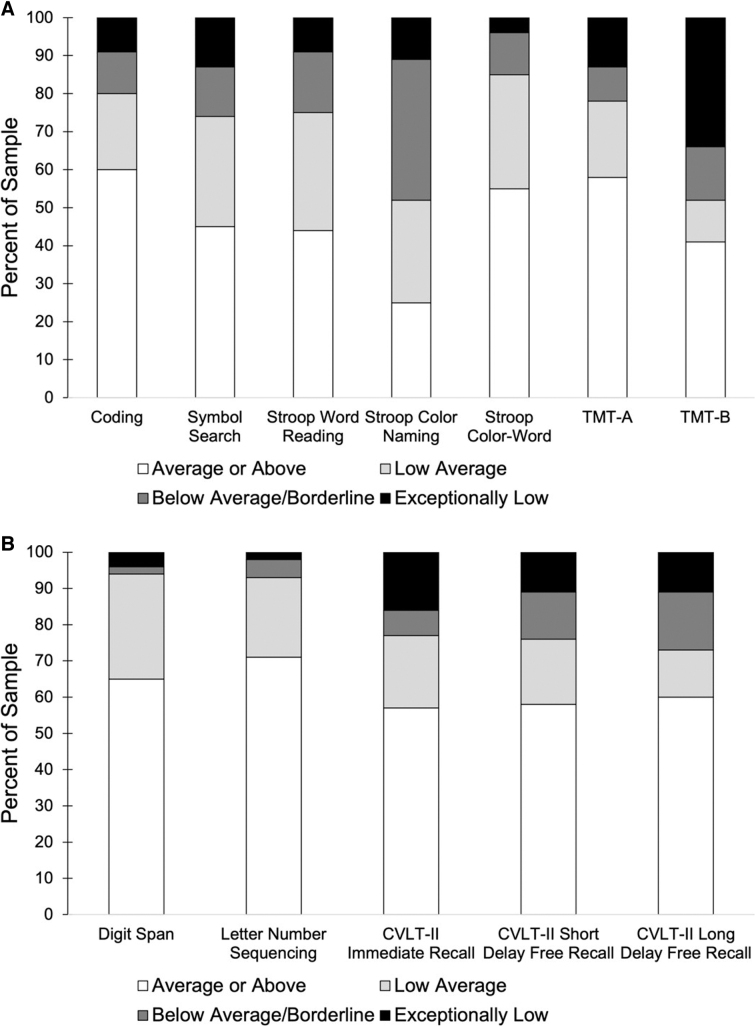
Percentage of the sample performing in the average or above, low average, below average/borderline, and exceptionally low range on each neuropsychological measure. Neuropsychological tasks are divided by measures of processing speed and executive function (**A**) and measures of working and episodic memory (**B**). CVLT-II, California Verbal Learning Test–Second Edition; TMT-A, Trail Making Test-A; TMT-B, Trail Making Test-A.

As shown in Figure 3, performance on individual neuropsychological measures varied, with generally worse performance on measures of processing speed and executive function than on measures of memory. A large percentage of the sample scored below the average range on TMT-B and Stroop Color Naming. TMT-B had the highest percentage of scores in the extremely low range (<1st percentile).

### Principal component analysis on neuropsychological battery

Variables entered into the PCA were raw scores on Digit Span Total, Letter Number Sequencing, Coding, Symbol Search, TMT-A, TMT-B, Stroop Word Reading, Stroop Color Naming, Stroop Color-Word, CVLT-II Immediate Recall, CVLT-II Short Delay Free Recall, and CVLT-II Long Delay Free Recall. After a varimax rotation of the PCA results, our KMO test of sampling adequacy demonstrated a strong relationship among the variables (KMO = 0.81). Bartlett's test of sphericity identified significance among the correlations within the correlation matrix (χ^2^_(66)_ = 374.483, *p* < 0.001).

The PCA yielded a two-component solution (i.e., two components with initial eigenvalues >1; Table 2). Component 1 mapped onto neuropsychological measures of processing speed and executive function and had an eigenvalue of 6.38 and accounted for 53.2% of the total variance. Component 2 mapped onto measures of working and episodic memory and had an eigenvalue of 1.68 and explained a further 14% of the variance. Varimax rotation led to a new eigenvalue of 4.43 for component 1 (processing speed and executive function) and 3.64 for component 2 (working and episodic memory; 36.9% and 30.3% of the variance, respectively). Rotated loadings of each neuropsychological measure on both components are shown in Table 2.

**Table 2.** Results of Principal Components Analysis: Rotated Component Matrix After Varimax Rotation

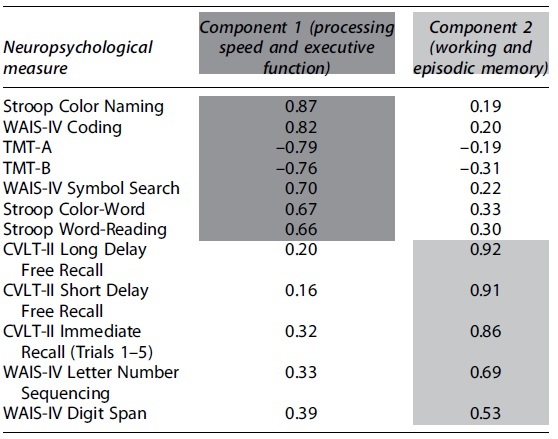

Shading depicts assignment (loading) of measures onto components based on the strength of correlation between individual neuropsychological measures and the components.

WAIS-IV, Wechsler Adult Intelligence Scale–Fourth Edition; TMT-A, Trail Making Test-A; TMT-B, Trail Making Test-B; CVLT-II, California Verbal Learning Test–Second Edition.

### Associations between the Attention Network Test, cognitive domain scores, and functional outcomes

**FIG. 4.**
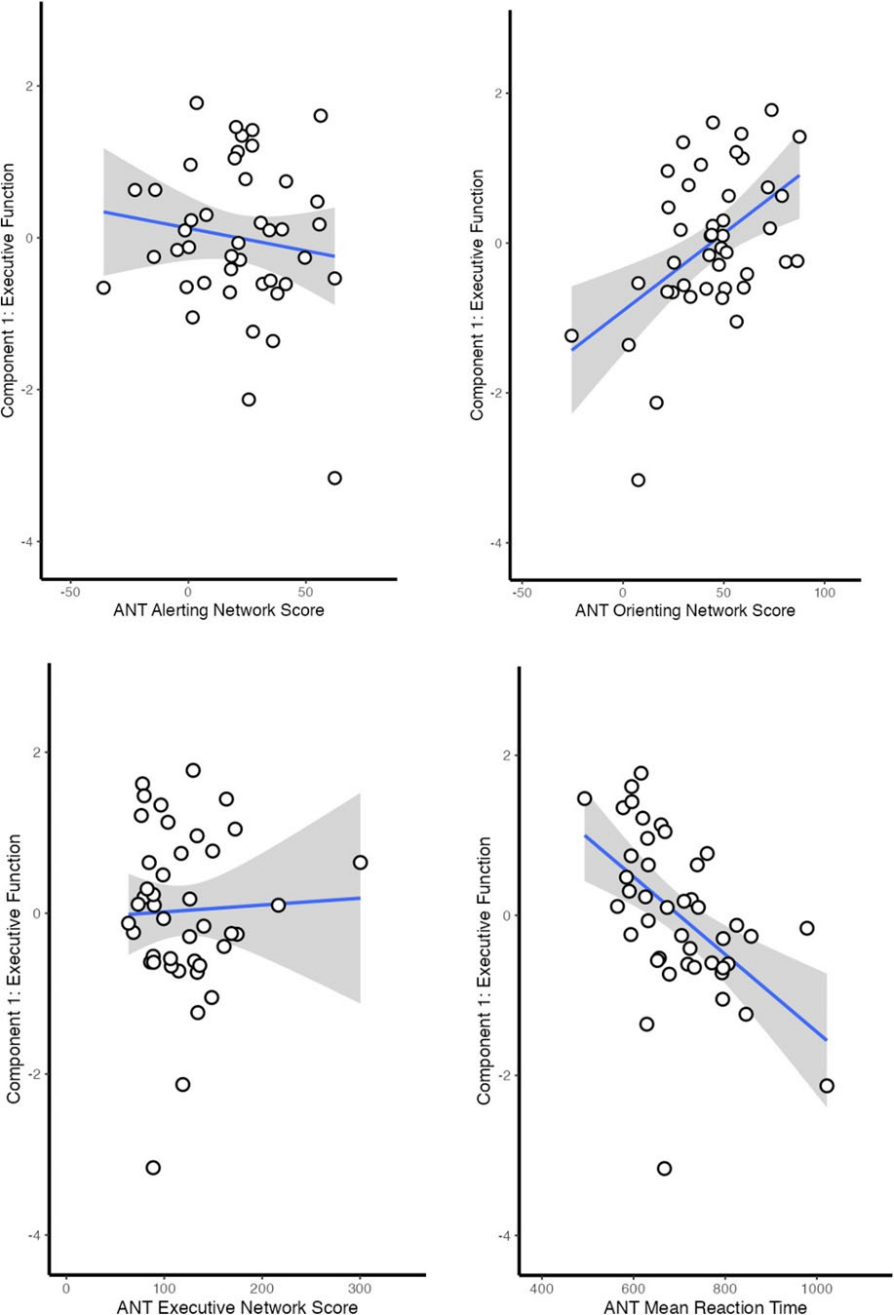
Scatter plots depicting the unadjusted association between ANT scores and component 1 (executive function). Shading represents the 95% confidence interval. ANT, Attention Network Test.

For the regression model with the outcome of component 1 (processing speed and executive function), overall regression with ANT performance was statistically significant (*R*^2^ = 0.67, *F*_(10,30)_ = 6.03, *p* < 0.001; Table 3A). The ANT executive network score was significant in the model (β = 0.26, *p* = 0.03), as was ANT mean RT (β = −0.49, *p* < 0.001). Figure 4 demonstrates the unadjusted correlations between ANT scores and component 1 as scatter plots.

**Table 3.** Regression Results with Outcome Variables of (A) Component 1 (Processing Speed and Executive Function); (B) Component 2 (Working and Episodic Memory); and (C) GOSE (A)

Model fit	R^2^	Adjusted* R*^2^	F	df	*p* value
	0.67	0.56	6.03	10, 30	<0.001

Parameters	Standardized coefficient* *(β)	95% CI lower	95% CI upper	t	*p* value
Age	–0.09	–0.39	0.21	–0.63	0.53
Education	0.30	0.02	0.57	2.20	0.04
Sex (female vs. male)	0.08	–0.46	0.61	0.29	0.77
Cause of injury (fall vs. vehicular accident)	−0.62	–1.15	–0.09	–2.38	0.02
Cause of injury (assault vs. vehicular accident)	–0.21	–0.83	0.41	–0.70	0.49
Cause of injury (other vs. vehicular accident)	–2.18	–3.73	–0.63	–2.87	0.007
ANT alerting	–0.07	–0.31	0.17	–0.58	0.57
ANT orienting	0.05	–0.30	0.40	0.30	0.77
ANT executive	0.26	0.02	0.51	2.22	0.03
ANT mean RT	–0.49	–0.76	–0.22	–3.72	<0.001

ANT, Attention Network Test; RT, reaction time; CI, confidence interval.

**Table:** (B)

Model fit	R^2^	Adjusted* R*^2^	F	df	*p* value
	0.34	0.11	1.51	10, 30	0.18

Parameters	Standardized coefficient* *(β)	95% CI lower	95% CI upper	t	*p* value
Age	0.34	–0.08	0.77	1.64	0.11
Education	0.08	–0.31	0.47	0.40	0.69
Sex (female vs. male)	0.66	–0.10	1.42	1.78	0.09
Cause of injury (fall vs. vehicular accident)	0.06	–0.69	0.81	0.17	0.86
Cause of injury (assault vs. vehicular accident)	–0.43	–1.31	0.44	–1.01	0.32
Cause of injury (other vs. vehicular accident)	0.73	–1.46	2.93	0.68	0.50
ANT alerting	0.05	–0.29	0.39	0.30	0.77
ANT orienting	0.03	–0.47	0.53	0.12	0.91
ANT executive	–0.01	–0.34	0.34	–0.01	0.99
ANT mean RT	–0.17	–0.55	0.21	–0.91	0.37

ANT, Attention Network Test; RT, reaction time; CI, confidence interval.

**Table:** (C)

Model fit	AIC	Nagelkerke R^2^	χ^2^	df	*p* value
	160	0.18	21.6	10	0.02

Parameters	Standardized coefficient* *(OR)	95% CI lower	95% CI upper	Z	*p* value
Age	1.01	0.96	1.06	0.47	0.64
Education	1.22	0.98	1.53	1.93	0.05
Sex (female vs. male)	0.51	0.12	2.17	–0.97	0.33
Cause of injury (fall vs. vehicular accident)	0.36	0.09	1.42	–1.46	0.15
Cause of injury (assault vs. vehicular accident)	0.37	0.06	2.05	–1.13	0.26
Cause of injury (other vs. vehicular accident)	0.02	0.01	0.62	–2.42	0.02
ANT alerting	1.01	0.98	1.03	0.72	0.47
ANT orienting	0.98	0.98	1.03	–1.50	0.13
ANT executive	1.02	1.003	1.03	2.51	0.01
ANT mean RT	0.99	0.99	1	–1.63	0.10

GOSE, Glasgow Outcome Scale-Extended; ANT, Attention Network Test; RT, reaction time; AIC, Akaike information criterion; OR, odds ratio; CI, confidence interval.

Overall regression for model 2 with the outcome of component 2 (working and episodic memory) and performance on the ANT was not statistically significant (*R*^2^ = 0.34, *F*_(10,30)_ = 1.51, *p* = 0.18; Table 3B). None of the ANT variables predicted scores on component 2.

A similar model using ordinal logistic regression was computed with GOSE scores as the outcome. Overall regression was statistically significant (Nagelkerke *R*^2^ = 0.18, *χ*^2^_(10)_ = 21.6, *p* = 0.02; Table 3C). Among specific predictors, the ANT executive network score weakly, but significantly, predicted GOSE score (odds ratio [OR] = 1.02, *p* = 0.01).

### Associations between the Attention Network Test and cognitive domain scores by injury severity

Given the relative preponderance of complicated mild TBI in our sample, we explored the association between the ANT and cognitive function by injury severity by recomputing regression models predicting the principal components, with separate models in complicated mild TBI and in moderate-severe TBI. In participants with complicated mild TBI, the regression model predicting component 1 (processing speed and executive function) remained significant (*R*^2^ = 0.74, *F*_(10,16)_ = 4.60, *p* = 0.003), with the executive network (*p* = 0.05) and mean response time (*p* = 0.04) significant in the model. The regression model predicting component 2 (memory) remained non-significant (*R*^2^ = 0.42, *F*_(10,16)_ = 1.13, *p* = 0.40). In participants with moderate-severe TBI, neither regression model was significant (component 1: *R*^2^ = 0.97, *F*_(10,1)_ = 3.00, *p* = 0.42; component 2: *R*^2^ = 0.90, *F*_(10,1)_ = 0.94, *p* = 0.67).

## Discussion

Our primary objective was to evaluate the relationship between the ANT and cognitive function in individuals with TBI. Our secondary objective was to assess the relationship between the ANT and global disability. ANT scores ranged from above to below age expectations, with a significant proportion of the sample performing below age expectation on mean RT. Neuropsychological scores varied, but were generally worse, on measures of processing speed and executive function than measures of memory. Global function on the GOSE also varied, but tended to cluster toward good recovery. PCA on the neuropsychological battery yielded two principal components, one comprising measures of processing speed/executive function and another comprising measures of memory. The ANT demonstrated a strong association with the processing speed/executive function component, but not the memory component. The ANT was a weak overall predictor of disability, although within ANT networks, the executive network showed an association with disability.

Consistent with our hypotheses, and with earlier findings in healthy adults, the present results demonstrated a moderately strong relationship between the ANT and a principal component assessing processing speed/executive function. Of the specific variables of the ANT, the executive network and mean RT demonstrated robust associations with this principal component. The association between the ANT executive network and processing speed/executive function may reflect the known role of the lateral pre-frontal cortex and anterior cingulate cortex in executive functions.^15,16,30–32^ This association would also be expected because the flanker paradigm used to compute the ANT executive score is closely tied to inhibition, a core executive function.^33^ Our results are consistent with past work that has suggested that the executive network may be more distinct from the alerting and orienting networks.^18,19^

That ANT mean RT was associated with the processing speed/executive function component—and more strongly than the ANT executive network—is not surprising given that the neuropsychological tests that comprised this component were response-time dependent and relied heavily on rapid information processing. This difference in the relative strength of association between ANT RT and the executive network may be reflective of the predominance of speeded tasks among the measures comprising this component, which included five measures of visual attention/processing speed and only two measures of higher-order executive functions (TMT-B and Stroop Color-Word). Executive function is an umbrella term for various higher-order cognitive processes, and it is possible that if our battery included additional executive measures (e.g., of organization, decision making) with a more balanced weighting with processing speed measures, we may have observed an even stronger association with the ANT executive network. It is notable that we did find an association, given the relatively small sample size of the study.

As hypothesized, the present findings did not support a strong association between the ANT and the principal component comprising memory. Inherently, the ANT is a behavioral measure developed to target the three aspects of attention and neural networks involved in attention and executive processes, rather than memory. Thus, the lack of association with memory provides additional evidence in support of the construct validity of the ANT and its specificity of association with clinical assessment of executive function in TBI.

We explored the association between the ANT and global function to further understand the clinical utility and ecological validity of the ANT. The current study highlights two findings related to this analysis: 1) The overall model shows weak association with global function, and 2) within specific networks, the ANT executive network is significantly associated with function, though in the opposite direction than was hypothesized. That the overall model had a weak association with global function is unsurprising given that neuropsychological measures themselves only have modest associations with daily function.^34^ Even perfect scores on the GOSE are associated with a wide variability in neuropsychological performance, which may explain the weak association we found between the GOSE and the ANT. The finding of a significant association between the ANT executive network and disability is consistent with research demonstrating that executive functions are strong predictors of activities of daily living and everyday function in healthy adults.^35,36^

Interestingly, we found that a stronger executive network (lower score on the ANT) was associated with greater disability (lower score on the GOSE). It is unclear why this pattern emerged in our data, though this association may be moderated by other variables and factors that we did not assess in our study, such as neuropsychiatric symptoms including depression or anxiety. Further research is necessary to understand this association and evaluate the ecological validity of the ANT.

### Limitations

Our sample was predominantly persons with complicated mild TBI and exploratory analyses evaluating the impact of injury severity suggested that the results were driven by persons with complicated mild TBI, but not persons with moderate-severe TBI. However, the number of persons with moderate-severe TBI was small, which risks a false negative (type 2 error), and thus caution is urged in generalizing findings to those with moderate-severe TBI until further evaluation is conducted. Our neuropsychological battery was limited with respect to the executive functions measured; however, all measures were chosen because of their sensitivity to deficits in TBI and to minimize participant burden. The neuropsychological battery did not include a measure of general intellectual function (IQ), which may have affected the results. The study was cross-sectional in nature, and future work will benefit from longitudinal follow-up to determine associations between the ANT, neuropsychological measures, and disability in the chronic stage of TBI. Finally, our sample size was relatively small, though it is notable that we found significant associations between the ANT and neuropsychological measures given this small sample.

## Conclusion

Findings support the construct validity of the ANT in subacute TBI and suggest that it may be a useful assessment measure in TBI clinical research. With future research, the ANT may eventually inform targeted interventions for persons with TBI by assisting in the identification of injury pathology, tracking engagement of the appropriate neuroanatomical targets (e.g., of non-invasive brain stimulation), and serving as a physiologically relevant cognitive end-point for clinical trials.^37^

## Transparency, Rigor, and Reproducibility Summary

The present study was not formally registered on ClinicalTrials.gov because it is not a clinical trial. The analytical plan was not pre-specified because this article constitutes a secondary analysis of a molecular imaging longitudinal study. Approximately 350 subjects were screened, and of those 48 enrolled in the study at the time of analysis, 3 subjects were excluded, resulting in our final sample of 45. Examiners were trained and supervised by a clinical neuropsychologist. All neuropsychological measures, as well as the global disability measure, are well validated, have strong psychometric properties, and are frequently used in TBI research. Examiners were not aware of the aims and hypotheses of this article. All data were securely stored in REDCap. Data can be made available upon reasonable request to the corresponding author and a subsequent data-use agreement.

## Abbreviations Used

ANT: Attention Network Test
CVLT-II: California Verbal Learning Test–Second Edition
GCS: Glasgow Coma Scale
GOSE: Glasgow Outcome Scale-Extended
KMO: Kaiser-Meyer-Olkin
OR: odds ratio
PCA: principal components analysis
RT: reaction time
SD: standard deviation
TBI: traumatic brain injury
TMT-A: Trail Making Test-A
TMT-B: Trail Making Test-B
WAIS-IV: Wechsler Adult Intelligence Scale–Fourth Edition
